# Effects of cyanobacterial extracellular products and gibberellic acid on salinity tolerance in *Oryza sativa *L

**DOI:** 10.1186/1746-1448-2-7

**Published:** 2006-06-06

**Authors:** AA Rodríguez, AM Stella, MM Storni, G Zulpa, MC Zaccaro

**Affiliations:** 1 Laboratory of Plant Physiology and Biology of Cyanobacteria. Department of Biodiversity and Experimental Biology; 2 Laboratory of Ecoporphyrin. Department of Biological Chemistry. Faculty of Exact and Natural Sciences. University of Buenos Aires, Pabellón II 4° Piso, Intendente Güiraldes 2620, Buenos Aires C1428EHA, Argentina

## Abstract

Salt stress is one of the most serious factors limiting the productivity of rice, the staple diet in many countries. Gibberellic acid has been reported to reduce NaCl-induced growth inhibition in some plants including rice. Most paddy soils have a natural population of Cyanobacteria, prokaryotic photosynthethic microorganisms, which synthesize and liberate plant growth regulators such as gibberellins that could exert a natural beneficial effect on salt stressed rice plants. The aim of this work was to evaluate the effect of the cyanobacterium *Scytonema hofmanni *extracellular products on the growth of rice seedlings inhibited by NaCl and to compare it with the effect of the gibberellic acid in the same stress condition. Growth (length and weight of the seedlings) and biochemical parameters (5-aminolevulinate dehydratase activity, total free porphyrin and pigments content) were evaluated.

Salt exposure negatively affected all parameters measured, with the exception of chlorophyll. Chlrorophyll concentrations nearly doubled upon exposure to high salt. Gibberellic acid counteracted the effect of salt on the length and dry weight of the shoot, and on carotenoid and chlorophyll b contents. Extracellular products nullified the salt effect on shoot dry weight and carotenoid content; partially counteracted the effect on shoot length (from 54% to 38% decrease), root dry weight (from 59% to 41% decrease) and total free porphyrin (from 31 to 13% decrease); reduced by 35% the salt increase of chlorophyll *a*; had no effect on root length and chlorophyll *b*. Gibberellic acid and extracellular products increased 5-aminolevulinate dehydratase activity over the control without salt. When coincident with high salinity, exposure to either EP or GA_3_, resulted in a reversal of shoot-related responses to salt stress. We propose that *Scytonema hofmanni *extracellular products may counteract altered hormone homeostasis of rice seedlings under salt stress by producing gibberellin-like plant growth regulators.

## Findings

Most paddy soils have a natural population of Cyanobacteria. Some representatives of these prokaryotic photosynthethic microorganisms are a potential source of combined nitrogen because they are capable of fixing atmospheric nitrogen [1]. An additional benefit of cyanobacteria is their capacity to synthesize and liberate bioactive substances such as auxins, gibberellins, cytokinins, vitamins, polypeptides, aminoacids, which promote plant growth and development [2-4].

Coastal salinity and accumulation of salts in irrigated land are primary factors depressing yield in rice crop. Salinity can affect germination, metabolism, the size of plants, branching, leaf size and overall plant anatomy. Salt also affects photosynthetic components such as enzymes, chlorophyll and carotenoid contents [5] as well as the activity of ALA-D [6], second enzyme of porphyrin biosynthetic pathway that produce heme group substances and chlorophyll in plants. The inhibitory effect of salt stress on plant growth is exhibited at several levels and involves an array of cellular processes such as cell division and expansion. These cellular processes are regulated by hormones for which homeostasis may be altered by salt. Several reports have indicated that application on crops of growth regulators, such as GA_3 _and cytokinin, produced some benefit in alleviating the adverse effects of salt stress [7]. GA_3 _reduced NaCl-induced growth inhibition of shoot rice seedlings [8], and kinetin lightened the influence of salinity on growth and production of plant growth regulators in *Vigna sinensis *and *Zea mays *[6].

The aim of this work was to evaluate the capacity of *Scytonema hofmanni *EP to counteract the NaCl-induced growth inhibition in rice seedlings and to compare it with the beneficial effect of gibberellic acid.

An axenic strain of the cyanobacterium *Scytonema hofmanni *(14 a), from the culture collection of the Laboratory of Cyanobacteria, University of Buenos Aires [9], was selected for this experiment because it had previously showed a promoting effect on the shoot length of rice seedlings in the same way as GA_3 _(Rodríguez, personal communication). *S. hofmanni *was cultured in W modified medium [9], under fluorescent light (45 μmol photon m^-2 ^s^-1^), photoperiod 12:12. At day 14 the cyanobacterial biomass was separated from the culture medium by centrifugation (40 min, 8000 × g, 10°C) under sterile condition. The productivity was 1.4 mg biomass DW/mL culture. The supernatant (EP) containing extracellular products, was sterilized by 0.22 μm pore size Millipore membrane and storaged at 4°C. Germinated sterile caryopses of *Oryza sativa *L. cv. Yeruá PA (kindly provided by La Arrocera Argentina SA) were cultivated in hidroponia. Plastic boxes containing 650 mL of the different culture media were placed under fluorescent light (45 μmol photon m^-2 ^s^-1^), photoperiod 12:12, 31 ± 1°C and 80 percentage humidity. The culture media were: W+5 g NaCl/L; W+5 g NaCl/L +0.5 mg GA_3_/L; EP+5 g NaCl/L and W (control). The concentration 5 g NaCl/L (85 mM) had been previously proved to inhibit all the parameters studied. We had also established that seedlings were most sensitive to salt at day 14. To avoid any effect of this strain capacity to fix nitrogen on the biological and biochemical parameters, from day 5, all treatments were added with 2 mL per day of the complete mineral solution of Hoagland, containing combined nitrogen [10]. At day 14, seedlings were harvested and shoot length, shoot dry weight (DW), root lenght and root DW were measured and the shoots were used for biochemical determinations, chlorophyll *a*, *b *and carotenoids, according to [11]; total protein, total free porphyrin and ALA-D (E.C. 4.2.1.24) activity according to [12]. One unit of ALA-D activity (U) was defined as the amount of enzyme which catalyzes the formation of 1 nmol of product/h under the standard incubation condition. The values obtained in a completely randomized design were analyzed by a one way-ANOVA and DGC test [13], using InfoStat statistical program 2002.

**Figure 1:**
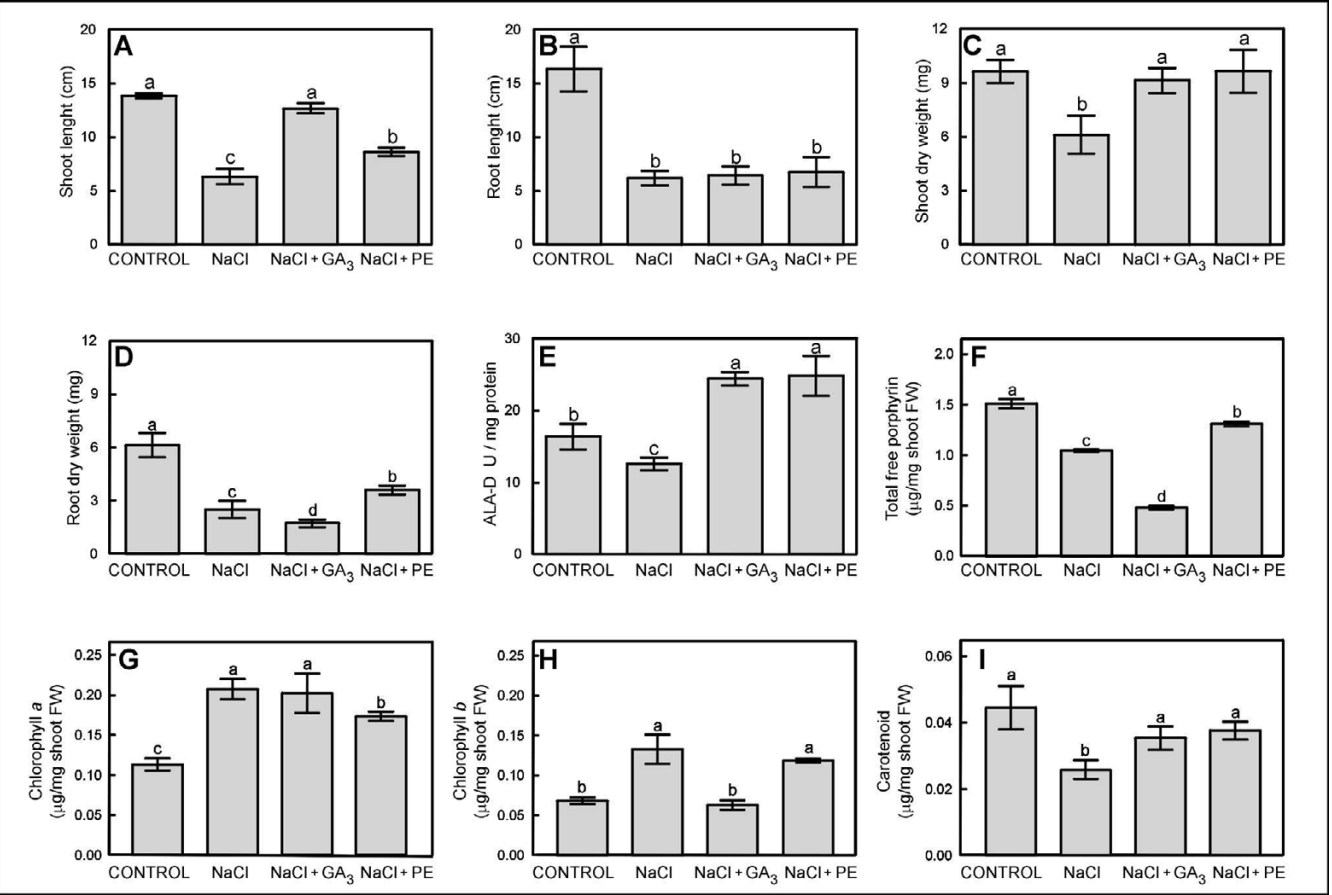
Effect of NaCl, NaCl+GA and NaCl+EP on rice seedling shoot length(A), root length (B), shoot dry weight (C) and root dry weight (D) rice shoot ALA-D activity (E), total free porphyrin (F), chlorophyll a (G) and b (H) and carotenoids (I) contents. Bars represent standard error. Different letters indicate significant difference, p < 0.05, n = 8.

Salt-exposed plant exhibited a reduction in shoot and root growth and biomass related to control plants. When coincident with high salinity, exposure to either EP or GA_3_, resulted in a reversal of shoot-related responses to salt stress but not to root responses. NaCl reduced shoot length by 54%, root length by 62%, shoot DW by 37% and root DW by 59% (Fig. 1A, B, C and 1D, respectively). This results coincide with other authors who worked with rice seedlings stressed by salt [8,14] established that the DW of plants decreased with salinity in two tolerant and sensitive rice cultivars.

GA_3 _counteracted the inhibition of shoot growth produced by salt (Fig. 1A and 1C). Lin and Kao [8] also established that salt-induced inhibition of rice shoot growth was reduced by GA_3_. In our experiment GA_3 _showed no difference with NaCl in the inhibition of root growth (Fig. 1B). GA_3 _produced an inhibition on root DW (72% compared with control), which is higher than the inhibition produced by salt (Fig. 1D).

EP partially counteracted NaCl-induced inhibition on shoot length (16% less inhibition) This result coincide with those obtained by inoculating Cyanobacteria in paddy fields affected by salt [15]. EP also increased root DW by 18% the salt value with salt (Fig. 1D). EP nullified the salt effect on shoot DW (Fig. 1C), but had no effect on the root growth inhibition by NaCl (Fig. 1B). GA_3 _is known to only affect shoot characters. GA_3 _and EP treatments alleviated the adverse salt effect on the shoot growth but were ineffective with respect to root growth. The coincident biological effects of GA_3 _and EP only on shoot growth indicate a possibility of EP containing gibberellin-like substances.

Salt exposure negatively affected all biochemical parameters with the exception of chlorophyll. Salt reduced ALA-D activity by 23% (Fig. 1E) This result coincide with other authors who also observed that, besides lowering ALA-D activity salt decreased GA_3 _content and increased ABA content [6]. Salt also reduced total free porphyrins by 31% (Fig. 1F), and carotenoids by 42% (Fig. 1I) but increased chlorophyll *a *and *b *by 83% and 96%, respectively (Fig. 1G and 1H). It was also observed in other rice cultivars that carotenoids were reduced by NaCl [5,16]. However, in other model systems, such as salt tolerant green algae, chlorophyll:carotenoids ratio tend to decrease as a function of salinity [17]. There are very few data available on the increase of chlorophyll content by salt. Pervaiz et al. demonstrated that chlorophyll content was progressively increased with the salinity in wheat [18].

GA_3 _and EP increased ALA-D activity alike, surpassing the control value by about 50% (Fig. 1E). Total free porphyrin was reduced by NaCl+GA_3 _by 70% comparing with the control without salt, so this parameter resulted in 55% reduction compared to NaCl treatment (Fig. 1F). GA_3 _did not counteract the effect of salt on chlorophyll *a *(Fig. 1G), but it did on chlorophyll *b *(Fig. 1H).

Compared to salt, EP increased total free porphyrin by 18% (Fig. 1F) and compared to the control without salt increased chlorophyll *a *content (Fig. 1G). With respect to chlorophyll *b*, EP did not produced a significant difference compared with salt (Fig. 1H). So, GA_3 _and EP produced similar effects on ALA-D activity and carotenoid content. EP partially reverted salt effect on total free porphyrin and increased chlorophyll a content; GA_3 _totally reverted the salt effect on the chlorophyll b content taking it to the control value. The decrease of carotenoid content produced by salt was totally counteracted by EP as well as by GA_3_.

In *Lupinus termis*, Haroun and Hussein [19] established that seed priming in cyanobacterial cultures filtrates increased *a *and *b *chlorophyll and reduced carotenoids content. Regarding plant growth regulators, this treatment also increased auxin, gibberellic acid and cytokinin content and decreased ABA content. There is a general agreement on the induction of the endogenous hormone levels by exogenous application of different growth regulators [20]. In our experiment the improvement of rice seedling salt tolerance was probably due to the presence of hormones in the EP produced by the cyanobacterium. Treatment with GA_3 _may have counteracted the excess of ABA produced as a response to the salt stress, reducing alterations in the growth phytoregulators ratios.

*S. hofmanni *EP reverted completely or partially many of the NaCl-induced effects on growth and also the biochemical alterations of Yerua PA rice seedlings. EP partially coincided with GA_3 _in the amelioration of salt affected parameters. The cyanobacterial EP would contain gibberellin-like substances which may be responsible of the alleviation of salt stress adverse effect on hormone homeostasis.

However, it would be important to confirm the presence of gibberellins in *S. hofmanni *extracellular products as well as the alteration of the hormonal homeostasis in this rice cultivar under salt stress.

## Abbreviations

Abscisic acid, ABA; aminolevulic acid dehidratase, ALA-D; dry weight, DW; extracellular products, EP; fresh weight, FW; gibberellic acid, GA_3_; indol acetic acid, IAA; Watanabe culture medium, W.

## Authors' contributions

AR studied the morphological and biochemical parameters and performed the statistical analysis.

AMS studied the biochemical parameters

MMS participated in the interpretation of data

GZ participated in the interpretation of data

MCZ, conception and design of the experiment, interpretation of data.

All the authors drafted, read and approved the final manuscript.
